# Gossypol Exhibits a Strong Influence Towards UDP-Glucuronosyltransferase (UGT) 1A1, 1A9 and 2B7-Mediated Metabolism of Xenobiotics and Endogenous Substances

**DOI:** 10.3390/molecules17054896

**Published:** 2012-04-27

**Authors:** Yong-Sheng Zhang, Jun Yuan, Zhong-Ze Fang, Yan-Yang Tu, Cui-Min Hu, Gan Li, Liang Wang, Jian-Ping Deng, Jia-Jiu Yao, Hai-Rong Li

**Affiliations:** 1 Tangdu Hospital, Fourth Military Medical University, Xi’an 710038, China; 2 Laboratory of Metabolism, Center for Cancer Research, National Cancer Institute, Bethesda, MD 20892, USA; 3 1001 Rockville Pike, Rockville, MD 20852, USA

**Keywords:** gossypol, UDP-glucuronosyltransferase (UGT), enzyme inhibition

## Abstract

Gossypol, the polyphenolic constituent isolated from cottonseeds, has been used as a male antifertility drug for a long time, and has been demonstrated to exhibit excellent anti-tumor activity towards multiple cancer types. The toxic effects of gossypol limit its clinical utilization, and enzyme inhibition is an important facet of this. In the present study, *in vitro* human liver microsomal incubation system supplemented with UDPGA was used to investigate the inhibition of gossypol towards UGT1A1, 1A9 and 2B7-mediated metabolism of xenobiotics and endogenous substances. Estradiol, the probe substrate of UGT1A1, was selected as representative endogenous substance. Propofol (a probe substrate of UGT1A9) and 3'-azido-3'-deoxythimidine (AZT, a probe substrate of UGT2B7) were employed as representative xenobiotics. The results showed that gossypol noncompetitively inhibits UGT-mediated estradiol-3-glucuronidation and propofol *O*-glucuronidation, and the inhibition kinetic parameters (K_i_) were calculated to be 34.2 and 16.4 μM, respectively. Gossypol was demonstrated to exhibit competitive inhibition towards UGT-mediated AZT glucuronidation, and the inhibition kinetic parameter (K_i_) was determined to be 14.0 μM. All these results indicated that gossypol might induce metabolic disorders of endogenous substances and alteration of metabolic behaviour of co-administered xenobiotics through inhibition of UGTs’ activity.

## 1. Introduction

**Figure 1:**
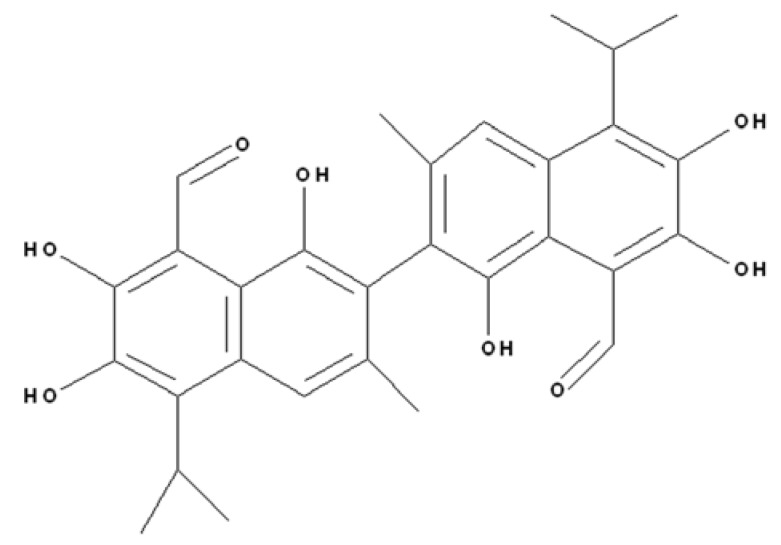
The structure of gossypol.

Gossypol (Figure 1), the polyphenolic constituent isolated from cottonseeds, has been used in China as a male antifertility drug for a long time. The excellent anti-tumor activities of gossypol have also drawn much attention. To date, gossypol has been reported to exhibit anti-cancer activities towards various types of tumors, such as Ehrlich ascites tumor cells [1], SW-13 adrenocortical carcinoma cells [2], hormone-dependent and hormone-independent breast carcinoma [3,4], colon carcinoma cell line HT-29 and LoVo [5]. Clinical research has been carried out for the anti-cancer application of gossypol. For example, the response rate of metastatic adrenal cancer to gossypol has been reported to be similar to other reagents currently used for adrenal cancer [6]. In the recent studies, the ability of gossypol to modulate prostate cancer has been widely investigated. The experiments performed by Volate *et al*. showed that gossypol can reduce the viability of prostate cancer cell lines (LAPC4, DU145 and PC-3) through inducing apoptosis [7]. Another study demonstrated that gossypol could induce autophagic cell death in apoptosis-resistant AI prostate cancer CL-1 and PC-3 cells [8].

The clinical application of gossypol has always been strongly limited by its toxicity. Gossypol has been reported to induce hepatotoxicity [9,10]. Administration of gossypol can result in pathological changes in rat and human testes and abnormal sperm [11]. The enzyme inhibition by gossypol is another toxicity problem. Gossypol has been demonstrated to inhibit some acrosomal enzymes of the rabbit sperm, including acrosin, azocoll proteinase, neuraminidase, and arylsulfatase [12]. Gossypol has been reported to inhibit rat hepatic microsomal enzymes [13].

UDP-glucuronosyltransferases (UGTs), membrane-bound drug metabolizing enzymes, can glucuronidate various endogenous and exogenous substances [14]. Inhibition of UGTs can result in the metabolic disorders of various endogenous and exogenous substances [15,16,17]. To date, the inhibitory effect of gossypol towards UGT-mediated metabolism of endogenous and exogenous substances remains unclear. Estradiol is a sex hormone and plays a key role in normal reproductive and sexual functioning. In human liver microsomes, estradiol is conjugated by UGTs to form either the corresponding 3- or 17-glucuronide. Estradiol-3-glucuronidation is primarily catalyzed in the liver by UGT1A1, with some involvement of UGT1A3. To date, the most widely used probe substrate for UGT1A1 has been estradiol. Propofol is a short-acting, intravenously administered hypnotic agent. Despite significant glucuronidation by extrahepatic UGT isoforms (UGT1A7, UGT1A8, UGT1A10), propofol is regarded as an appropriate probe for UGT1A9 in human liver. 3'-azido-3'-deoxythimidine (AZT), frequently prescribed to patients infected with the human immunodeficiency virus, can rapidly undergo glucuronidation after absorption. The major involved UGT isoform is UGT2B7 [18].

The aim of the present study is to investigate the inhibition of gossypol towards UGT1A1, UGT1A9 and UGT2B7-mediated metabolism of xenobiotics and endogenous substances. The human liver microsome incubation system was used and UGT1A1-catalyzed estradiol 3-glucuronidation (E3G) reaction, UGT1A9-catalyzed propofol glucuronidation and UGT2B7-catalyzed 3'-azido-3'-deoxythimidine (AZT) glucuronidation were selected as the probe reactions.

## 2. Results and Discussion

**Figure 2:**
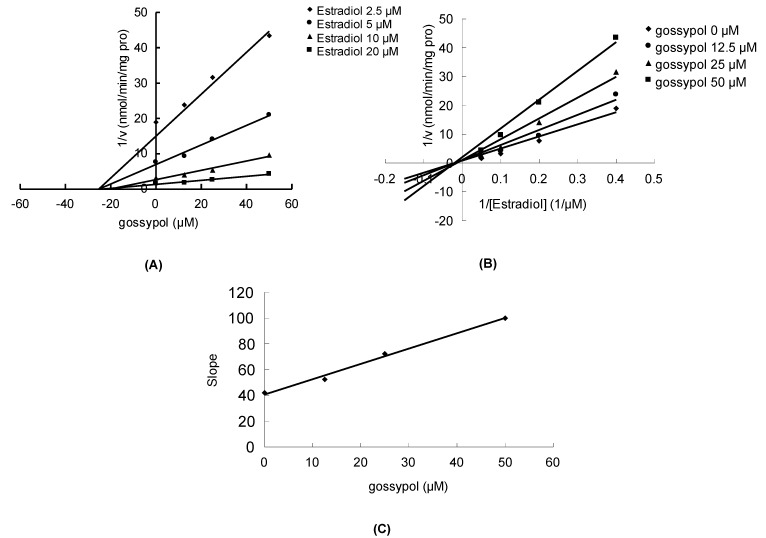
The inhibition of gossypol towards UGT-mediated estradiol glucuronidation. (**A**) Dixon plot of the inhibitory effect of gossypol on UGT-mediated estradiol glucuronidation; (**B**) Lineweaver-Burk plot of the inhibitory effect of gossypol on UGT-mediated estradiol glucuronidation; (**C**) Second plot of slopes from Lineweaver-Burk plot *vs*. gossypol concentrations. All the experiments were carried out in duplicate.

**Figure 3:**
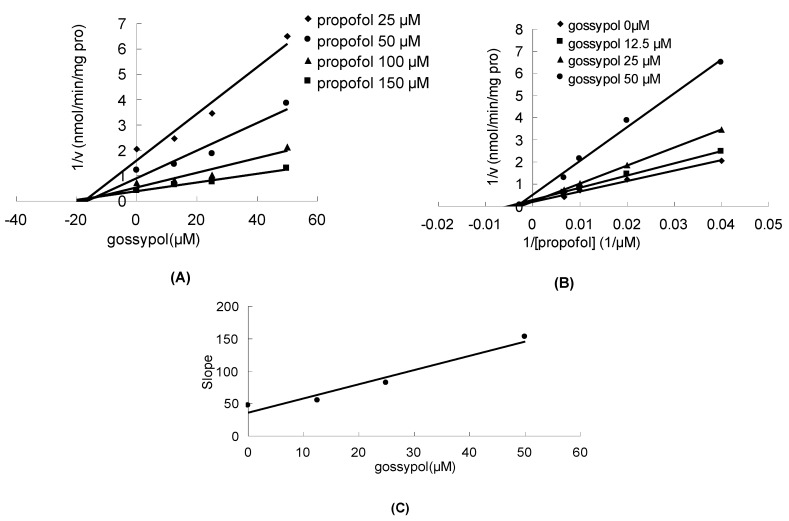
The inhibition of gossypol towards UGT-mediated propofol glucuronidation. (**A**) Dixon plot of the inhibitory effect of gossypol on UGT-mediated propofol glucuronidation; (**B**) Lineweaver-Burk plot of the inhibitory effect of gossypol on UGT-mediated propofol glucuronidation; (**C**) Second plot of slopes from Lineweaver-Burk plot *vs*. gossypol concentrations. All the experiments were carried out in duplicate.

**Figure 4:**
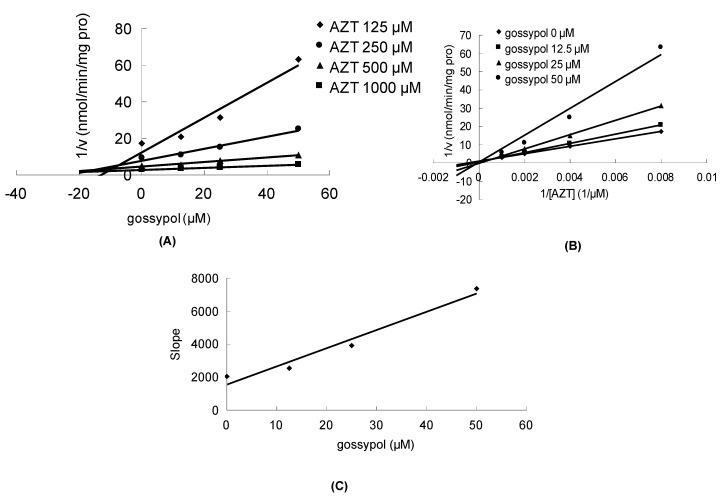
The inhibition of gossypol towards UGT-mediated AZT glucuronidation. (**A**) Dixon plot of the inhibitory effect of gossypol on UGT-mediated AZT glucuronidation; (**B**) Lineweaver-Burk plot of the inhibitory effect of gossypol on UGT-mediated AZT glucuronidation; (**C**) Second plot of slopes from Lineweaver-Burk plot *vs*. gossypol concentrations. All the experiments were carried out in duplicate.

Gossypol can significantly inhibit the metabolism of estradiol, propofol and AZT in a concentration-dependent manner, with corresponding IC_50_ values of 23.5 ± 0.7, 32.4 ± 0.7 and 28.5 ± 2.1 μM, respectively. As shown in Figure 2A,B, the Dixon plot and Lineweaver-Burk plot indicated that gossypol noncompetitively inhibited the metabolism of estradiol in HLM incubation system. The inhibition kinetic parameter (K_i_) was calculated to be 34.2 μM (Figure 2C). Similarly, gossypol inhibited the metabolism of propofol in the noncompetitive manner, as indicated by Dixon plot (Figure 3A) and Lineweaver-Burk plot (Figure 3B). Second plot using slope of Lineweaver-Burk plot *vs*. gossypol concentration showed that the inhibition kinetic parameter (K_i_) was 16.4 μM (Figure 3C). Different from the inhibitory type for the metabolism of estradiol and propfol, the inhibition of the metabolism of AZT metabolism was best fit to competitive inhibition type (Figure 4A,B), and the inhibition kinetic parameter (Ki) was determined to be 14.0 μM (Figure 4C).

The inhibition of enzymes by gossypol has been drawing the attention of researchers for a long time. The experiments carried out by Smit *et al.* demonstrated that gossypol could inhibit the activity of catechol-O-methyltransferase (COMT) which is a key enzyme located in nerve cells and liver, and involved in the metabolism of catecholamines [19]. Gossypol was demonstrated to selectively inhibit the type 1 steroid 5a-reductase isoform which catalyzed the NADPH-dependent reduction of the double bond of a variety of 3-oxo-∆^4^ steroids, including the conversion of testosterone to 5a-dihydrotestosterone. Glutathione-S-transferases (GSTs) are a family of metabolic enzymes mainly found in the cytosol of hepatic, renal and intestinal cells, which catalyse the conjugation of electrophilic substances to glutathione. Both the gossypol enantiomers were demonstrated to exhibit equipotent inhibition towards GST-μ isoenzymes, but (−)-gossypol was more potent against the GST-α isoenzymes [20]. Gossypol has also been reported to exert influence towards endoplasmic and mitochondrial cytochrome P450s which are the most important drug-metabolizing enzymes [21]. The renal and hepatic toxicities of gossypol have been attributed to its interaction with the iron of the P450 enzyme.

In the present study, attention was paid to the inhibitory effect of gossypol on the one of most important phase II enzymes UGTs. Gossypol was demonstrated to exhibit inhibitory effects on the metabolism of xenobiotic and endogenous substances catalyzed by UGT1A1, 1A9 and 2B7, with the Ki values of 34.2, 16.4 and 14 μM, respectively. Previous reports have showed that the maximum plasma concentration can reach more than 10 μg/mL (19.3 μM) after mice received a single 50 mg/kg intravenous dose [22], which is very close to Ki values and suggest the risk of metabolic disorders of some endogenous substances and alteration of metabolic behaviour of co-administered xenobiotics through inhibition of UGTs’ activity.

## 3. Experimental

### 3.1. Chemicals and Reagents

Gossypol was granted from Biaffin GmbH & Co KG (Kassel, Germany). Estradiol, propofol, 3'-azido-3'-deoxythimidine (AZT), alamethicin, 4-methylumbelliferone (4-MU), 4-methyl- umbelliferone-β-D-glucuronide (4-MUG), Tris-HCl, and uridine 5'-diphosphoglucuronic acid (UDPGA) (trisodium salt) were purchased from Sigma-Aldrich (St. Louis, MO, USA). All other reagents were of HPLC grade or of the highest grade commercially available. Human liver microsomes (HLMs) were purchased from BD Gentest Corp. (Woburn, MA, USA).

### 3.2. Human Liver Microsome (HLM) Incubation System

Gossypol was dissolved in DMSO at 20 mmol/L as stock solution. A typical incubation mixture (200 μL total volume) contained HLMs (final concentration: 0.5 mg/mL), 5 mM UDPGA, 5 mM MgCl_2_, 50 mM Tris-HCl buffer (pH 7.4), 50 μg/mg protein alamethicin, and the selective substrates. Incubations with the selective substrates were performed at the concentration corresponding to the apparent K_m_ or S_50_ value reported for each isoform. The incubation time was 30 min. Determination of half inhibition concentration (IC_50_) of gossypol was carried out as previously described [15,16]. When determining the inhibition kinetic type and parameters, various concentration of gossypol and probe substrates (estradiol for UGT 1A1 at 2.5, 5, 10, 20 μM; propofol for 1A9 at 25, 50, 100, 150 μM; 3'-azido-3'-deoxythimidine (AZT) for 2B7 at 125, 250, 500, 1,000 μM) was used. The analysis of samples were carried out as previously reported [23,24,25]. In brief, glucuronide metabolites of estradiol and propofol were separated using mobile phases consisting of 10% methanol (Solvent A), 100% methanol (Solvent B), and 30% acetonitrile with 2 mM perchloric acid (Solvent C). Initial HPLC solvent conditions were 85% Solvent A and 15% Solvent C. Solvent C was held constant at 15% during the HPLC run. Metabolites were eluted by a linear increase in Solvent B overa 20-minute period. UV detection was at 280 nm for estradiol glucuronides, and 215 nm for the propofol glucuronide. For the analysis AZT glucuronidation, the HPLC column was eluted at 1 mL min^−1^ with a mobile phase of acetonitrile:aqueous (v/v = 12:88). The aqueous phase contained 0.4 mL concentrated H3PO4 diluted to 1 l with water (pH 2.4). Ultraviolet detection was at 267 nm.

### 3.3. Data Analysis and Statistics

The results were expressed as mean ± standard deviation (SD). Statistical differences were evaluated using the two-tailed Student’s *t*-test.

## 4. Conclusions

Gossypol can noncompetitively inhibit the UGT1A1-mediated estradiol glucuronidation and UGT1A9-mediated propofol metabolism, and competitively inhibit UGT2B7-mediated AZT glucuronidation. These results indicated that gossypol might induce metabolic disorders of endogenous substances and alteration of metabolic behaviour of co-administered xenobiotics through inhibition of UGTs’ activity.
